# Resequencing analysis of five Mendelian genes and the top genes from genome-wide association studies in Parkinson’s Disease

**DOI:** 10.1186/s13024-016-0097-0

**Published:** 2016-04-19

**Authors:** Bruno A. Benitez, Albert A. Davis, Sheng Chih Jin, Laura Ibanez, Sara Ortega-Cubero, Pau Pastor, Jiyoon Choi, Breanna Cooper, Joel S. Perlmutter, Carlos Cruchaga

**Affiliations:** Department of Internal Medicine, School of Medicine, Washington University, 8007, 660 South Euclid Avenue, St. Louis, MO 63110 USA; Department of Neurology, School of Medicine, Washington University, St. Louis, MO USA; Department of Psychiatry, School of Medicine, Washington University, St. Louis, MO USA; Department of Neurology, Complejo Asistencial Universitario de Palencia, Palencia, Spain; Center for Applied Medical Research (CIMA) University of Navarra School of Medicine, Pamplona, Spain and Centro de Investigación Biomédica en Red Enfermedades Neurodegenerativas (CIBERNED), Madrid, Spain; Movement Disorders Unit, Department of Neurology, University Hospital Mutua de Terrassa, University of Barcelona, Terrassa, Barcelona Spain; Department of Radiology, Anatomy & Neurobiology, Program in Occupational Therapy, Program in Physical Therapy, Washington University, St. Louis, MO USA; Hope Center Program on Protein Aggregation and Neurodegeneration, Washington University, St. Louis, MO USA

**Keywords:** Parkinson’s, Association study, *SNCA*, *LRRK2*, *PARKIN*, *PINK1*, *DJ-1*, *MAPT*, *GBA rare variants, gene-based analysis*

## Abstract

**Background:**

Most sequencing studies in Parkinson’s disease (PD) have focused on either a particular gene, primarily in familial and early onset PD samples, or on screening single variants in sporadic PD cases. To date, there is no systematic study that sequences the most common PD causing genes with Mendelian inheritance [*α-synuclein* (*SNCA*), *leucine-rich repeat kinase 2* (*LRRK2)*, *PARKIN*, *PTEN-induced putative kinase 1* (*PINK1*) and *DJ-1 (Daisuke-Junko-1)*] and susceptibility genes [*glucocerebrosidase beta acid* (*GBA*) and *microtubule-associated protein tau* (*MAPT*)] identified through genome-wide association studies (GWAS) in a European-American case-control sample (n=815).

**Results:**

Disease-causing variants in the *SNCA,LRRK2* and *PARK2* genes were found in 2 % of PD patients. The *LRRK2*, p.G2019S mutation was found in 0.6 % of sporadic PD and 4.8 % of familial PD cases. Gene-based analysis suggests that additional variants in the *LRRK2* gene also contribute to PD risk. The *SNCA* duplication was found in 0.8 % of familial PD patients. Novel variants were found in 0.8 % of PD cases and 0.6 % of controls. Heterozygous Gaucher disease-causing mutations in the *GBA* gene were found in 7.1 % of PD patients. Here, we established that the *GBA* variant (p.T408M) is associated with PD risk and age at onset. Additionally, gene-based and single-variant analyses demostrated that *GBA* gene variants (p.L483P, p.R83C, p.N409S, p.H294Q and p.E365K) increase PD risk.

**Conclusions:**

Our data suggest that the impact of additional untested coding variants in the *GBA* and *LRRK2* genes is higher than previously estimated. Our data also provide compelling evidence of the existence of additional untested variants in the primary Mendelian and PD GWAS genes that contribute to the genetic etiology of sporadic PD.

## Background

PD is the second most common neurodegenerative disorder after Alzheimer’s disease (AD) [1]. By the year 2030, the prevalence of PD is projected to be between 8.7 and 9.3 million [1]. Genetic studies in PD have provided valuable insights into the underlying pathogenic mechanisms [2], leading to the development of animal models for investigation of disease mechanisms and identification of novel therapeutic targets [3]. Initial studies of multiplex families with PD found concordance rates of 75 % in monozygotic twins, 22 % in dizygotic twins [5], and an increased relative risk of PD of 2.9 (95 % CI 2.2–3.8) for those with an affected first-degree relative [6]. These findings indicate that the genetic etiology of PD does not fit a simple genetic model [5]. GWAS of PD have identified variants at 20 loci influencing PD risk [2, 4, 7–9], with population-specific differences [10, 11]. The currently identified genetic factors explain only 6–7 % of the phenotypic variability associated with PD [12], and the most prevalent GWA signals account for only 3–5 % of PD genetic variance in individuals of European ancestry [12]. These results provide unequivocal, compelling evidence for the existence of undiscovered genetic factors that contribute to the etiology of PD. Both candidate gene association studies and GWAS repeatedly validate that the most statistically significant signals associated with PD are common variants located close to *SNCA*, *LRRK2*, *MAPT* genes and low frequency coding variants in the *GBA* gene [2, 4, 7, 10, 13–16].

Non-coding variants are the most significant single nucleotide polymorphisms (SNPs) identified near the *MAPT* and *SNCA* genes by GWAS [4]. To date, the functional variants driving such associations are unknown. We hypothesize that low frequency or rare coding variants can be identified by re-sequencing the *MAPT* and *SNCA* genes. In addition, deep-sequencing *LRRK2* and *GBA* genes can not only identify additional untested coding risk variants but also protective alleles, as previously reported in these genes [17].

Highly penetrant mutations in the *SNCA* and *LRRK2* genes are found in families with autosomal dominant inheritance, whereas autosomal recessive families with a typical PD phenotype carry mutations in the *PARK2/PARKIN*, *PARK6*/*PINK1* and *PARK7/DJ-1* genes [18]. Most genetic studies in PD have focused on sequencing a particular Mendelian gene in familial or early onset PD, or have directly screened few variants in sporadic PD cases in small samples [18]. A systematic study that sequences all of these genes (*SNCA, LRRK2, PARK2, PINK1* and *PARK7*) in a large PD dataset has not been reported in European Americans [19, 20]. Thus, we used next-generation sequencing technology to re-sequence five Mendelian and the top GWAS susceptibility PD genes in a well-characterized case–control European American dataset (478 cases and 337 healthy controls) to identify both risk and protective low frequency or rare variants for PD.

## Results

We performed pooled DNA-targeted deep-sequencing of the protein-coding regions of 7 genes, including 5 genes previously reported to most frequently cause familial forms of PD (*SNCA, LRRK2, DJ-1, PARK2 and PINK1)* and 2 genes that have significant associations in GWAS with sporadic PD (*GBA and MAPT* genes) in 478 PD patients and 337 healthy individuals of European-American descent from the Washington University in Saint Louis Movement Disorder Clinic (Table 1) [15, 21]. This cohort contains 83 % late-onset PD (LOPD) and 74 % sporadic PD cases.

**Table 1:** Summary of the sample demographics

Series	n	Age at onset mean ± SD (range) in years	Age at clinical assessment mean ± SD (range) in years	Male: Female ratio	Caucasian (%)
Total PD	478	61.3 ± 10.6 (40–86)	67.6 ± 10.0 (41–90)	297:181	99
Familial PD	126	59.0 ± 10.9 (40–85)	65.8 ± 11.0 (42–86)	73:53	89
EOPD	83	44.68 ± 2.95 (38–49)	53.4 ± 6.6 (43–71)	51:32	99
Control	337		64.8 ± 10.2 (30–85)	117:220	92

### Rare variants in a European American case-control sample

We validated missense and splice-affecting variants with a predicted minor allele frequency (MAF) <5 %. In this European-American descent sample, a total of 47 low-frequency (0.5–5 %) and rare (<0.5 %) non-synonymous coding variants were validated. 36.2 % (17/47) of the variants are found in *LRRK2*, 21.2 % (10/47) in *GBA*, 17 % (8/47) in *PARK2*, 14.9 % (7/47) in *PINK-1*, 8.5 % (4/47) in *MAPT* and 2.1 % (1/47) in *DJ-1* (Table 2). 70 % of these variants are either singletons (24/47) or doubletons (9/47).

**Table 2:** Summary of the variants found in the European-American case-control sample

Gene	AA change	Cases (478)	MAF	Controls (337)	MAF	*p* value	Clinical interpretation PD mutation database	Notes
LRRK2	R50H	0	0	1	0.001	n.s.	Unknown	Autosomal Dominant
	R521G	0	0	1	0.001	n.s.	Unknown	
	R793M	0	0	2	0.003	0.09	Pathogenic nature unclear	
	S885C	1	0.001	0	0	n.s.	Novel	
	L119P	2	0.002	1	0.001	n.s.	Non-pathogenic	
	P1262A	1	0.001	0	0	n.s.	Non-pathogenic	
	I1371V	2	0.002	0	0	n.s.	Pathogenic nature unclear	
	V1389I	1	0.001	0	0	n.s.	Unknown	
	V1450I	0	0	1	0.001	n.s.	Not pathogenic	
	R1514Q	7	0.007	4	0.006	n.s.	Not pathogenic	
	M1646T	21	0.022	9	0.013	n.s.	Not pathogenic/Risk	
	L1795F	1	0.001	0	0	n.s.	Pathogenic nature unclear	
	D1887N	1	0.001	0	0	n.s.	Novel	
	G2019S	8	0.008	0	0	0.02	Pathogenic	
	N2081D	17	0.018	15	0.022	n.s.	Non-pathogenic	
	Y2189C	0	0	1	0.001	n.s.	Pathogenic nature unclear	
	A2461V	0	0	1	0.001	n.s.	Unknown	
Total		62		36				
DJ-1	A179T	1	0.0010	0	0	n.s.	Pathogenic nature unclear	Autosomal Recessive
Total		1		0				
PARKIN	D53X	1	0.001	0	0	n.s.	Pathogenic	Autosomal Recessive
	R65C	1	0.001	1	0.001	n.s.	Pathogenic nature unclear	
	A82E	1	0.001	1	0.001	n.s.	Pathogenic nature unclear	
	R275W	2	0.002	2	0.003	n.s.	Pathogenic nature unclear	
	E310D	0	0	1	0.001	n.s.	Pathogenic nature unclear	
	R402C	5	0.005	1	0.001	n.s.	Pathogenic nature unclear	
	R402H	0	0	1	0.001	n.s.	Unknown	
	P437L	3	0.003	2	0.003	n.s.	Pathogenic nature unclear	
Total		13		9				
PINK1	R147C	0	0	1	0.001	n.s.	Novel	Autosomal Recessive
	R207Q	1	0.001	0	0	n.s.	Unknown	
	M318L	0	0	1	0.001	n.s.	Pathogenic nature unclear	
	A339S	2	0.002	1	0.001	n.s.	Pathogenic nature unclear	
	N367S	2	0.002	0	0	n.s.	Pathogenic nature unclear	
	G411S	1	0.001	0	0	n.s.	Pathogenic nature unclear	
	R492X	0	0	1	0.001	n.s.	Pathogenic	
Total		6		4				
GBA^c^	R83C	2	0.002	0	0	n.s.	Unknown	PD GWAS Hit
	H294Q	2	0.002	0	0	n.s.	Pathogenic	
	T336S	1	0.001	0	0	n.s.	Novel	
	E365K	19	0.020	11	0.02	n.s.	Polymorphism, Risk PD	
	T408M	17	0.018	0	0	0.0005	Polymorphism	
	N409S	7	0.007	1	0.001	0.09	Pathogenic, Risk PD	
	E427K	1	0.001	0	0	n.s.	Unknown	
	D448H	1	0.001	1	0.001	n.s.	Pathogenic^a^	
	L483P	7	0.007	2	0.003	n.s.	Pathogenic^a^, Risk PD	
	A495P	17	0.018	10	0.015	n.s.	Pathogenic^b^	
Total		74		25		0.001		
MAPT	A152T	4	0.004	0	0	0.09	Risk AD, FTD	PD GWAS Hit
	S427F	0	0	2	0.003	0.09	Unknown	
	A495T	0	0	1	0.001	n.s.	Non-pathogenic	
	A556T	1	0.001	0	0	n.s.	Unknown	
Total		5		3				

Gene: official Symbol provide by HGNC; AA Change: amino acid change resulting from the observed variant; MAF: Minor allele frequency; Clinical Interpretation: Clinical interpretation is based on PD mutation database [22] and published papers. ^a^GBA variants found in pseudo gene. ^b^ Variant also known as p.A485P ^c^ Amino acid designations are based on the primary GBA translation product, including the 39-residue signal peptide

### Novel variants

8.5 % (4/47) of the total variants are novel and not present in public databases (accessed on June 11th, 2015). All of the novel singleton variants located on *LRRK2*, p.D1887N and p.S885C, and *GBA*, p.T336S genes are present exclusively in LOPD patients (Table 3). The *PINK1* p.R147C, variant was found in one control individual but was not present in public datasets.

**Table 3:** Summary of the individuals with novel variants in PD genes

Gene	AA	Location (Chr:bp)	ID	Ethnicity	AAO	PD FAM HISTORY	Gender	Dementia	MMSE
PINK1	R147C	1:20964386	1	Caucasian	NA	NO	F	NA	NA
LRRK2	S885C	12:40681305	2	Caucasian	67	NO	F	Yes	28
LRRK2	D1887N	12:40722164	3	Caucasian	55	NO	M	No	30
GBA	T336S	1:155206254	4	Caucasian	62	NO	F	Possible	27

### Copy number analysis

**Fig. 1:**
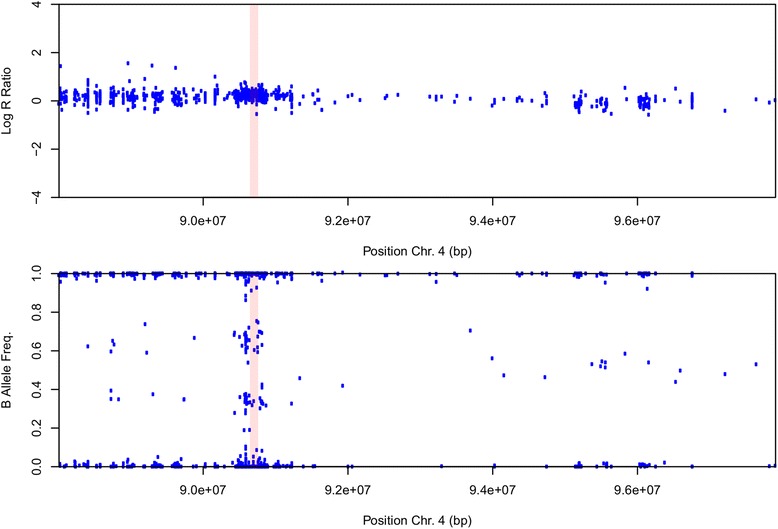
SNCA duplication. The lower panel shows genotyping data from PD patient, generated using NeuroXchip. Shown is B Allele frequency for each single-nucleotide polymorphism (SNP) assayed, in which a value of 0 indicates a homozygous A/A genotype, a value of 1 indicates a homozygous B/B genotype, and a value of 0.5 represents a heterozygous A/B genotype. The highlighted region (pink) delimits the duplicated segment; within this region are a lack of heterozygous calls and clusters of points at a B allele frequency of ∼ 0.33 and ∼ 0.66, which, coupled with an increased log R ratio (*upper panel*), are indicative of A/A/B and A/B/B genotype calls, respectively. Figure plotted using R

We observed a single structural genomic variant in a 70-year-old man with a family history of PD (1/126; 0.8 %; Fig. 1). B allele frequency and log R ratio indicate that this variant is an intra-chromosomal duplication at the *SNCA* locus. We did not identify this duplication, or any duplication at this locus, in control individuals. No other exonic rearrangements were observed in any PD patient in the *PARK2*, *DJ-1* or *PINK-1* loci.

### Known pathogenic variants

91.5 % (43/47) of the validated variants are reported in the PD mutation database [22]. Among the previously known variants, 7 % (3/43) are considered Mendelian pathogenic mutations for PD (*LRRK2* p.G2019S, *PINK1* p.R492X and *PARK2* p.D53X) (Table 4). Six out of eight *LRRK2* p.G2019S carriers reported PD family history. Thus, in this sample, 0.6 % (2/352) of the sporadic PD patients and a 4.8 % (6/126) of the familial PD subjects carry the *LRRK2* p.G2019S mutation. The *PARK2* p.D53X mutation heterozygous carrier is an EOPD patient with a positive family history. The *PINK1* p.R492X heterozygous carrier is an asymptomatic 68-year old individual with no family history of PD (Table 4).

**Table 4:** Summary of the individuals with pathogenic mutations in PD genes

Gene	AA	rs#	ID	Ethnicity	AAO	PD FAM HISTORY	Gender	Dementia	MMSE
LRRK2	G2019S	rs34637584	1	Caucasian	42	YES	M	No	30
			2	Caucasian	62	YES	F	No	30
			3	Caucasian	63	YES	M	No	30
			4	Caucasian	68	YES	M	Yes	16
			5	Caucasian	70	YES	M	No	30
			6	Caucasian	77	NO	M	Yes	25
			7	Caucasian	51	YES	F	Possible	23
			8	Caucasian	59	NO	F	No	29
PARK2	D53Stop	6:162864360	9	Caucasian	50	YES	M	Possible	28
PINK1	R492X	rs34208370	10	Caucasian	NA	NO	M	Possible	24

Of all the previously known variants in all sequenced genes, 11.6 % (5/43) are located in the *GBA* gene (p.H294Q, p.D448H, p.N409S, p.L483P and p.A495P) and cause Gaucher disease (Table 2). We found that these variants are overrepresented in the PD patient sample, but did not reach statistical significance (*p* = 0.08; OR = 1.76, 95 % CI = 0.93–3.34). Two *GBA* variants, p.T408M and p.E365K, previously described as non-pathogenic polymorphisms for Gaucher disease, are significantly enriched (*p* = 0.01; OR = 2.35, 95 % CI = 1.19–4.66) in PD patients (7.5 %; 36/478) compared with controls (3.2 %; 11/337).

### Variants of unclear and unknown pathogenicity

34.9 % (15/43) of the variants located on *LRRK2* (four variants), *PARK2* (six variants), *PINK1* (4 variants) and *PARK7* (one variant) have been reported previously and their pathogenicity is unclear (Table 2). Although the cumulative frequency of these variants is higher in PD patients (4.4 %) compared to controls (3.8 %), this difference is not statistically significant (*p* = 0.7; OR = 1.14, 95 % CI = 0.56–2.32), suggesting that either most of these variants are very unlikely to be true risk factors for PD or our sample size is not large enough to detect such differences.

There are 10 variants (21.3 %) with an unknown role in PD. In this cohort, 1.2 % of PD patients and 1.8 % of controls were found to carry one of these variants (*p* = 0.5; OR = 0.70, 95 % CI = 0.22–2.19).

The non-pathogenic variants, constituting 16.3 % (7/43) of the variants, were found in a similar proportion of PD patients (10 %) and controls (9.2 %) (*p* = 0.68; OR = 1.10, 95 % CI = 0.68–1.77), supporting their role as non-pathogenic.

### Single-variant analysis

The minor allele of *GBA* p.T408M (*p* = 4.9 × 10^−4^) is associated with increased PD risk after multiple-testing correction (Table 2). The *GBA* p.T408M variant is present in 3.5 % (17/478) of the total number of PD cases and in none of the control group (Table 2). In addition, we found a nominal association with *LRRK2* p.G2019S (*p* = 0.02) (Table 2). Using publicly available data from the exome variant server (EVS, European American) and Exome Aggregation Consortium (ExAc, European non-Finish) as controls, the variants p.T408M (*p* = 9.0 × 10^−4^; *p* = 1.0 × 10^−2^), p.L483P (*p* < 1.0 × 10^−4^; not found in ExAc), p.R83C (*p* = 1.0 × 10^−2^; *p* = 1.0 × 10^−4^), p.N409S (*p* = 2.0 × 10^−2^; *p* = 6.0 × 10^−2^), p.H294Q (*p* = 4.0 × 10^−2^; *p* = 1.0 × 10^−2^) and p.E365K (*p* = 4.0 × 10^−2^; *p* = 2.0 × 10^−2^) in the *GBA* gene, p.G2019S (*p* < 1.0 × 10^−4^; *p* < 1.0 × 10^−4^) and p.M1646T (not found in EVS; *p* = 3.0 × 10^−2^) in the *LRRK2 gene* and *PINK1* p.N367S (Not found in EVS; *p* = 1.0 × 10^−4^) all achieved statistical significance in at least one of the control populations studied (Table 5).

**Table 5:** Frequency of validated variants in public databases

Gene	AA change	MAF PD patients	EVS MAF	*p* value	OR (IC 95 %)	ExAC MAF	*p* value	OR (IC 95 %)
LRRK2	G2019S	0.008	0.0006	<0.0001	12.67 (4.0–40.2)	0.00063	<0.0001	13.4 (6.2–28.6)
	M1646T	0.022	0.0154	n.s.		0.01424	0.03	1.58 (1.02–2.45)
PINK1	N367S	0.002	Not found			0.00002	0.0001	139.6 (12.6–1541)
GBA	H294Q	0.002	0.0003	0.04	6.0 (1.0–35.99)	0.0004	0.01	5.82 (1.37–24.7)
	R83C	0.002	0.0001	0.01	18.02 (1.63–199)	7.5086E-05	0.0001	27.9 (5.4–144)
	N409S	0.007	0.0028	0.02	2.63 (1.13–6.13)	3.6300E-03	0.06	2.03 (0.95–4.31)
	L483P	0.007	0.0005	<0.0001	15.85 (4.63–54.24)	Not found		
	T408M	0.018	0.0072	0.0009	2.49 (1.45–4.28)	0.010	0.01	1.85 (1.14–3.01)
	E365K	0.020	0.0121	0.04	1.65 (1.01–2.71)	0.012	0.02	1.69 (1.06–2.67)
MAPT	A152T	0.004	0.0027	0.4	1.56 (0.54–4.54)	0.002	0.07	2.5 (0.92–6.82)

The *MAPT* p.A152T variant has been associated with other neurodegenerative diseases including AD and frontotemporal dementia (FTD) [23]. In our study, the *MAPT* p.A152T variant occurs in 0.8 % (4/478) of PD cases but in none of the controls (0/337, *p* = 0.09).

### Gene-burden analyses

To determine whether rare variants in the *LRRK2, DJ1, PARK2, PINK1, GBA* or *MAPT* genes contribute collectively to PD risk, we performed a gene-burden association test using the optimal SNP-set sequence kernel association test (SKAT-O) [24]. Gene-based association testing achieved significance for *GBA* (PSKAT-O = 7.0 × 10^−4^; OR = 2.28 (1.41–3.68). Importantly, the most commonly reported *GBA* risk variants (p.N409S and p.L483P) occur in 2.9 % (14/478) of the PD cases and in 0.9 % (3/337) of the controls (*p* = 0.05; OR = 3.35, 95 % CI = 0.95–11.8). When we exclude p.N409S and p.L483P from the analysis, the role of *GBA* in PD risk remains significant (*p* = 4.9 × 10^−3^; OR = 2.04, 95 % CI = 1.24–3.37), suggesting that additional variants in this gene also increase risk for PD. When we exclude p.T408M from the analysis, the risk of PD conferred by *GBA* variants is not significant (*p* = 0.39), which suggests that p.T408M may be the primary driver of the association with PD risk. These findings highlight the importance and necessity to sequence the entire *GBA* gene as opposed to genotyping only known risk variants for PD.

We also found a significant enrichment of coding variants in the *LRRK2* gene in PD cases compared to controls (*p* = 0.01, OR = 1.86, 95 % CI = 1.14-3.02) (Table 6), which suggests that there are other risk variants in the *LRRK2* gene in addition to the known pathogenic p.G2019S mutation.

**Table 6:** Gene-based analyses for Mendelian and GWAS PD genes

Gene	cMAF PD cases	cMAF controls	P	OR
GBA	0.084	0.034	0.0007	2.28 (1.41–3.68)
LRRK2	0.069	0.034	0.01	1.86 (1.14–3.02)
DJ-1	0.001	0	0.64	2.1 (0.08–52.1)
PARKIN	0.014	0.012	0.96	1.01 (0.43–2.40)
PINK1	0.006	0.005	0.93	1.05 (0.29–3.77)
MAPT	0.005	0.004	0.82	1.17 (0.27–4.95)

Results of SKAT-O analyses including all the validated coding variants were presented. cMAF = cummulative MAF

No significance was found for the *MATP2, PARKIN, PINK1* and *DJ-1* genes

### Effect on age at onset (AAO) of PD

**Fig. 2:**
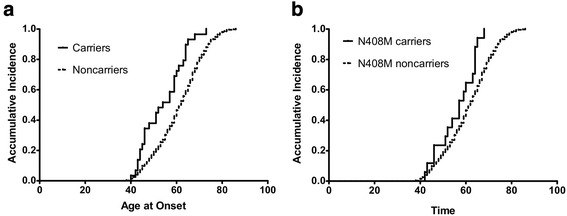
**a.** Cumulative incidence rates of PD among carriers and non-carriers of all *GBA* variants. **b.** Cumulative incidence rates of PD among carriers and non-carriers of the p.N408M variant. Survival fractions were calculated using the Kaplan-Meier method and significant differences were calculated by Log-rank test

*GBA* variant carriers tend to exhibit an earlier AAO than non-carriers [25]. Thus, we tested whether *GBA* variants affect AAO; we found that *GBA* variants carriers have a earlier AAO than non-carriers (54 years. vs. 62 years.; *p* < 0.0001) (Fig. 2a). Interestingly, when restricted to carriers and non-carriers of p.N408M using the same model, carriers had a 5.0-year-earlier onset than non-carriers (57 years. vs. 62 years.; *p* = 0.006) (Fig. 2b).

## Discussion

Disease-causing variants in the *SNCA, LRRK2, PARKIN, PINK1* and *DJ-1* genes have been found in familial early onset forms of PD [18]. In this study, we systematically screened for rare variants and pathogenic mutations in the *SNCA, LRRK2, PARK2, PINK1, PARK7, MAPT* and *GBA* genes in a series of well-characterized PD case-control samples. A total of 47 low-frequency and rare non-synonymous coding variants were validated.

### Most common pathogenic variants in this cohort

Nine individuals (1.9 %) of the total sample of PD patients carry a known pathogenic mutation in two Mendelian genes, *LRRK2* p.G2019S and *PARK2* p.D53X. Among patients with a family history of PD, 5.6 % (7/126) carry a known pathogenic mutation. In this cohort, we found that among the sequenced genes, the *LRRK2* gene was enriched with multiple variants, accounting for 36.2 % of all the validated variants. The *LRRK2* p.G2019S mutation is significantly associated with risk of PD and occurs in 1.7 % of PD patients. Interestingly, mutation carriers were clinically indistinguishable from idiopathic PD, which support the evidence for involvement of this gene in late-onset sporadic PD. A recent meta-analysis reported that the mean frequency of the *LRRK2* p. G2019S mutation in sporadic PD patients among studies in the U.S. is 0.4 % [26]. Meanwhile, another international multi-center study reports only 49 of 8371 (0.6 %) PD patients of European and Asian origin carry the *LRRK2* p. G2019S mutation [17]. Both frequencies are similar to the frequency reported here of 0.6 % (2/352) in sporadic PD patients. Our gene-based analysis found a significant association with the *LRRK2* gene, which suggests that there are additional risk variants in *LRRK2* affecting PD risk.

We also detected a *SNCA* locus duplication in a 70-year-old man with a family history of PD (1/126; 0.8 %; Fig. 1) and a 3-year history of parkinsonism. This PD patient exhibited clinical features indistinguishable from idiopathic PD. As expected, we found no coding mutations in the *SNCA* gene in this cohort. Point mutations in the *SNCA* gene are extremely rare and have been identified mostly in familial and EOPD [18]. The most common variation found in the *SNCA* gene are copy number variations (CNVs). *SNCA* duplications are not fully penetrant and are associated with variable clinical features, ranging from early-onset with dementia and psychiatric features to late-onset sporadic [27].

A recent report examining rare variants in the main Mendelian PD genes in a small case–control sample consisting of 249 cases and 145 controls of European origin (Spanish) found an enrichment of rare functional variants in PD cases [20]. They reported that up to 3.6 % of patients with sporadic PD are carriers of known pathogenic mutations in different Mendelian genes. The difference in the frequency of pathogenic mutations reported here (1.9 %) and that reported by Spataro (3.6 %) [20] is likely due to differences in methodology (exome sequencing data vs pooled-targeted sequencing) and to the different genetic background of the samples (Spanish vs North American).

### Most common risk variants in this cohort

Heterozygous mutations in the *GBA* gene can be considered as low penetrance variants with autosomal dominant inheritance for PD [28]. In this study, fifty-three (11 %) of the PD patients and fifteen (4.5 %) controls carry heterozygous variants in the *GBA* gene (*p* = 1.0 × 10^−3^; OR = 2.17, 95 % CI = 1.36–3.46), which indicate that *GBA* coding variants increase risk for PD in this cohort. We also have demonstrated that those patients with PD carrying a *GBA* variant experience a disease onset 6 years earlier than patients without *GBA* variants. Interestingly, *GBA* variants mainly affect AAO of LOPD patients. Two *GBA* variants (p.N409S and p.L483P) have consistently been reported to be associated with increased PD risk in both, Ashkenazi Jewish and non-Ashkenazi populations [29]. Here, the p.N409S (MAF = 0.007) and p.L483P (MAF = 0.007) variants, are present in 2.9 % (14/478) of PD patients and 0.9 % (3/337) of controls. These allelic frequencies agree with previous reports [29]. We found that both variants are overrepresented in PD cases compared to controls, but they only reached statistical significance after including a larger control sample from publicly available databases. In addition, we report for the first time, an association between PD risk and the *GBA* variant p.T408M (MAF: 0.018). p.T408M is considered a polymorphism because it has been found in control populations [25, 30]. In this dataset, the *GBA* p.T408M variant drives the gene-based association with risk for PD. In the largest Non-Ashkenazi case–control sample studied to date, the *GBA* p.T408M variant was not significantly associated with PD [29]. This discrepancy could be explained by the heterogeneity of populations included in that study as it was enriched with individuals from populations in which the p.T408M variant is absent or very rare. The p.E365K allele is a hypomorphic variant (42.7 % of wild type activity) [31] often found in *cis* or *trans* with other Gaucher-causing non-synonymous mutations [32], exhibiting a frequency that is similar in controls and Gaucher patients [33]. We found that p.E365K achieves nominal significance (*p* = 0.02; OR = 1.69, 95 % CI = 1.06–2.67) after including controls from public databases. Interestingly, the OR found here is similar to those reported previously [33, 34]. Both p.T408M and p.E365K have been described as “mild” mutations or modifier alleles. In our study, we did not observe a “second” mutation that occurred with either p.T408M or p.E365K, which suggests a second hit may exist as an interacting factor, similar to those described in a traditionally considered non-pathogenic variant in AD [35]. Interestingly, we found seven PD patients carrying PD risk variants in two of all screened genes, further suggesting a double-hit mechanism impacting the risk for PD (Table 7), as reported by the presence of variants in the *LRRK2* and *GBA* genes in PD patients [36].

**Table 7:** Individuals carrying two rare variants

Individual	Ethnicity	AAO	PD FAM HISTORY	Gender	Age at draw	rs#	Variant in GBA	rs#	Second Hit	Both genes
PD Patient	Caucasian	56	NO	F	66	rs71653622	A179T	rs2230288	E365K	GBA and PARK7
Control	Caucasian	NA	NO	F	65	rs421016	L483P	rs1064651	D448H	GBA and GBA
PD Patient	Caucasian	46	NO	F	57	rs76763715	N409S	rs75548401	T408M	GBA and GBA
PD Patient	Caucasian	63	NO	M	77	rs1141812	R83C	rs33995883	N2081D	GBA and LRRK2
PD Patient	Caucasian	82	NO	M	87	rs368060	A495P	rs33995463	L119P	GBA and LRRK2
Control	Caucasian	NA	NO	M	66	rs421016	L483P	rs35658131	Y2189C	GBA and LRRK2
PD Patient	Caucasian	59	YES	F	61	rs76763715	N409S	rs33995883	N2081D	GBA and LRRK2
PD Patient	Caucasian	73	NO	M	75	rs76763715	N409S	rs33995883	N2081D	GBA and LRRK2
Control	Caucasian	NA	NO	F	65	rs368060	A495P	rs34424986	R275W	GBA and PARK2
PD Patient	Caucasian	44	NO	M	50	rs76763715	N409S	rs45478900	G411C	GBA and PINK1

We also found that the *MAPT* p.A152T variant occurs in 0.8 % (4/478) of PD cases but in none of the controls (0/337, *p* = 0.09). It is possible that the *MAPT* p.A152T variant increases PD risk, but this association needs further confirmation in additional series.

Among the eight variants validated in *PARK2*, we found a stop-codon, p.D53X, in an EOPD (early onset PD) patient with a family history of PD. We also found one control individual carried the *PINK1* (p.R492X) variant. We validated just a single variant *DJ-1*, p. A179T in a 56 year old PD patient with no family history of PD. All of these variants in recessive genes were found a heterozygous manner. Truly causative variants in *PARK2, PINK1 or DJ-1* are present in a homozygous or heterozygous compound manner, but we cannot exclude the possible role of heterozygous variants on risk of sporadic PD. It is important to highlight that the most common pathogenic mutations in these genes are exon rearrangements or copy number variations. We did not detect exonic rearrangements in these genes in our cohort. The high proportion (83 %) of LOPD and sporadic cases (74 %) in our sample may explain the low number of validated variants found in the recessive genes.

### Novel variants

We uncovered four novel variants (*LRRK2*, p.D1887N and p.S885C), (*PINK1, p.R147C),* and (*GBA*, p.T336S) in 0.8 % of PD cases. *LRRK2,* p.D1887N is located in the kinase domain and could play a functional role. The rareness of and the impossibility to expand the segregation studies with these variants to additional family members make its clinical interpretation challenging. However, finding novel variants in sporadic late onset PD suggests that it is possible to uncover such variants in genes linked to Mendelian PD or even in PD cases with an unclear pattern of inheritance. This is supported by our gene-based analysis, which demonstrates that additional untested variants in the *GBA* and *LRRK2* genes contribute to the role of these genes in PD risk.

## Conclusions

In summary, our results confirm the strong effect of *GBA* and *LRRK2* on sporadic PD risk. However, our gene-based analyses demonstrates that non-synonymous *GBA* variants can have a greater impact on PD risk than *LRRK2*. In this cohort, the more common pathogenic mutations are located in the *LRRK2* gene. Multiple *GBA* gene variants confer the highest risk for PD in our sample. We report novel interactions between variants in the *GBA* and *LRRK2* genes as double hits affecting PD patients with no family history of PD. Our results also suggest that novel and untested variants in the *GBA* and *LRRK2* genes influence PD risk. This has important implications on the genetic information provided to patients and families and potential new therapeutic approaches for PD patients. Our findings also strongly support the role of the lysosomal system as a pathogenic pathway in PD. Further work is necessary to clarify the role of specific and very rare variants in these genes on risk and PD phenotype.

## Methods

### Ethics statement

The Institutional Review Board (IRB) at the Washington University School of Medicine in Saint Louis approved the study. Prior to their participation, written informed consent was reviewed and obtained from family members. The Human Research Protection Office (HRPO) approval number for our ADRC Genetics Core family studies is 201104178.

### Samples

Samples included 478 PD patients and 337 healthy individuals from the Washington University in Saint Louis Movement Disorder Clinic (MO, USA) [15, 21, 37]. All were examined by experienced movement disorder clinicians (J.S.P.). PD diagnosis was established according to the UK Brain Bank criteria.

### Statistical and association analyses

For each variant, allele frequencies were calculated in cases and controls, and a *χ*2 test on allelic association was performed. A *p*-value of 0.05 was set as nominal significance threshold. The multiple-testing correction cutoff for the single-variant analysis using Bonferroni correction for 47 tests is 1.0 × 10^−3^ (0.05/47). We used Plink (http://pngu.mgh.harvard.edu/~purcell/plink/) to analyze associations [38]. The gene-based association was performed using SKAT-O, which utilizes the R package SKAT [24]. All variants were included in the model independent of their clinical interpretation. The influence of the genetic variants on AAO was carried out using the Kaplan-Meier method and tested for significant differences using a log-rank test.

### Pooled-DNA sequencing experiment

Pooled-DNA sequencing was performed as described previously [35, 39, 40]. Briefly, equimolar amounts of individual DNA samples were pooled together after being measured using Quant-iT PicoGreen reagent. Two different pools with 100 ng of DNA from 114 and 98 individuals were made. The coding exons and flanking regions (a minimum of 50 bp each side) were individually PCR amplified using specific primers and Pfu Ultra high-fidelity polymerase (Stratagene). An average of 20 diploid genomes (approximately 0.14 ng DNA) per individual were used as input for a total of 62 PCR reactions that covered 46,319 bases from the 7 genes. PCR products were cleaned using QIAquick PCR purification kits, quantified using Quant-iT PicoGreen reagent and ligated in equimolar amounts using T4 Ligase and T4 Polynucleotide Kinase. After ligation, concatenated PCR products were randomly sheared by sonication and prepared for sequencing on an Illumina Genome Analyzer IIx (GAIIx) according to the manufacturer’s specifications. pCMV6-XL5 amplicon (1908 base pairs) was included as a negative control. As positive controls, ten different constructs (p53 gene) with synthetically engineered mutations at a relative frequency of one mutated copy per 250 normal copies were amplified and pooled with the PCR products. Six DNA samples heterozygous for previously known mutants in *MAPT* gene were also included. Single reads (36 bp) were aligned to the human genome reference assembly build 36.1 (hg18) using SPLINTER [41]. SPLINTER uses the positive control to estimate sensitivity and specificity for variant calling. The wild type: mutant ratio in the positive control is similar to the relative frequency expected for a single mutation in one pool (1 chromosome mutated in 125 samples = 1/250). SPLINTER uses the negative control (first 900 bp) to model the errors across the 36-bp Illumina reads and to create an error model from each sequencing run of the machine. Based on the error model, SPLINTER calculates a *p*-value for the probability that a predicted variant is a true positive. A *p*-value at which all mutants in the positive controls were identified was defined as the cut-off value for the best sensitivity and specificity. All mutants included as part of the amplified positive control vector were found upon achieving >30-fold coverage at mutated sites (sensitivity = 100 %) and only ∼ 80 sites in the 1908 bp negative control vector were predicted to be polymorphic (specificity = ∼95 %). The variants with a *p*-value below this cut-off value were considered for follow-up confirmation.

### Genotyping

All rare missense or splice site variants identified by SPLINTER were validated by directly genotyping all sequenced individuals using Sequenom iPLEX or KASPar genotyping systems as described previously [42–44]. The validated SNPs were then genotyped in all members of the series. An average coverage of 30-fold per allele per pool is the minimum coverage necessary to obtain an optimal positive predictive value for the SNP-calling algorithm [41]. The necessary number of lanes to obtain a minimum of 30-fold coverage per base and sample were run.

### Copy number variation analysis

The B Allele frequency and Log R Ratio were used to identify genomic deletions and duplications as previously described [45] using NeuroX chip data [46].

### Bioinformatics

The PD mutation database [22] was used to identify sequence variants previously found in other studies of familial PD and to determine whether or not they are considered to be disease-causative variants. The EVS (http://evs.gs.washington.edu/EVS/), SeattleSeq Annotation (http://snp.gs.washington.edu/SeattleSeqAnnotation137/), The Exome Aggregation Consortium (ExAC) http://exac.broadinstitute.org/ (June 19, 2015) and the Ensembl Genome Database (http://useast.ensembl.org/index.html) were used to annotate the rare variants. Polyphen algorithms were used to predict the functional effect of the identified variants.

### Population structure

A PCA was conducted to infer genetic structure of individuals who have GWAS data available using the EIGENSTRAT software as previously described [40]. Samples were excluded if not located within the EA cluster. Individuals who do not have GWAS data available were included in the study if the self-reported ethnicity was non-Hispanic European.

## Abbreviations

AAO: age at onset
AD: Alzheimer’s disease
CI: confidence interval
CNVs: copy number variations
DJ-1: Daisuke-Junko-1
EOPD: early-onset Parkinson’s disease
EVS: exome variant server
ExAc: exome aggregation consortium
FTD: frontotemporal dementia
GBA: glucocerebrosidase beta acid
GWAS: genome-wide association studies
LOPD: late-onset Parkinson’s disease
LRRK2: leucine-rich repeat kinase 2
MAF: minor allele frequency
MAPT: microtubule-associated protein tau
OR: odd ratio
PD: Parkinson’s disease
PINK1: PTEN-induced putative kinase 1
SKAT-O: SNP-set sequence kernel association test
SNCA: α-synuclein
